# Effects of lung cancer cell-associated B7-H1 on T-cell proliferation *in vitro* and *in vivo*

**DOI:** 10.1590/1414-431X20165263

**Published:** 2016-06-20

**Authors:** K. Chen, H.T. Huang, W.J. Hang, L.B. Pan, H.T. Ma

**Affiliations:** Department of Cardiothoracic Surgery, the First Affiliated Hospital of Soochow University, Jiangsu, China

**Keywords:** Costimulatory molecule, B7-H1, A549, Lewis lung carcinoma, Immunosuppression, T-cell proliferation

## Abstract

B7 homolog 1 (B7-H1) is the most potent immunoinhibitory molecule in the B7 family. In this study, we examined the effects of tumor-associated B7-H1 on T-cell proliferation in lung cancer. The expression of B7-H1 in human adenocarcinoma A549 and mouse Lewis lung carcinoma (LLC) cells were examined by flow cytometry. To assess the *in vitro* effect of tumor-associated B7-H1 on T-cell proliferation, we isolated T cells from peripheral blood mononuclear cells (PBMCs) of healthy individuals, labeled them with carboxyfluorescein succinimidyl ester, and co-cultured them with A549 cells in the absence or presence of anti-B7-H1 antibody. For *in vivo* analysis, LLC cells were subcutaneously injected into mice treated or not with anti-B7-H1 antibody. T-cell proliferation in both *in vitro* and *in vivo* assays was analyzed by flow cytometry. *In vitro,* co-culturing T cells with A549 cells significantly inhibited the proliferation of the former compared with the proliferation of T cells alone (P<0.01), and the addition of B7-H1 blocking antibody dramatically reversed the inhibition of T-cell proliferation by A549 cells. Similarly, in mice bearing LLC-derived xenograft tumors, *in vivo* administration of anti-B7-H1 antibody significantly increased the total number of spleen and tumor T cells compared to levels in control mice that did not receive anti-B7-H1 antibody. Functionally, *in vivo* administration of anti-B7-H1 antibody markedly reduced tumor growth. Tumor-associated B7-H1 may facilitate immune evasion by inhibiting T-cell proliferation. Targeting of this mechanism offers a promising therapy for cancer immunotherapy.

## Introduction

The immune system critically regulates cancer development. Extensive studies support the presence of cancer immunoediting, a process that evolves through three sequential phases during tumor development: elimination, equilibrium, and escape (1,2). During the elimination phase, immune cells recognize and destroy newly transformed cells, preventing the development of cancer, as supported by the susceptibility of immunodeficient or gene-targeted mice to spontaneous or carcinogen-induced tumors (2). In the equilibrium phase, a het-erogeneous population of tumor cells reaches a dynamic balance with the host immune system, with the presence of tumor cells detected but net tumor growth kept under control. This is often the longest phase of cancer immunoediting that allows tumor cells to accumulate further immunoevasive mutations, and the occurrence of this phase has been extensively proved in a variety of cancers (3). In the final escape phase, cancer cells acquire sufficient and specific genetic and/or epigenetic alterations that overcome the host immunocompetence through two major mechanisms: *i*) tumor cell-autonomous alterations that evade immune detection, and *ii*) tumor cell-induced modification in immune cells to generate an immunosuppressive microenvironment (4,5). Biologically, immunoevasion is considered a hallmark of cancer (6). Therefore, understanding the molecular mechanisms underlying each step of immunoediting will foster the rational design of immunotherapies targeting cancer.

B7 homolog 1 (B7-H1), also known as programmed death ligand-1 or CD274, is a member in the B7 immunoregulatory molecules. It is a cell-surface glycoprotein widely, yet minimally expressed on normal tissues and organs, and up-regulated in response to inflammation on cells including CD4^+^, CD8^+^ T cells, dendritic cells, macrophages, B cells, regulatory T cells (Tregs), epithelial cells, and endothelial cells (7). Furthermore, high levels of B7-H1 expression have been extensively reported in multiple human malignancies including lung, breast, ovarian, cervical, oral, head and neck, brain, gastric, liver, colorectal, nasopharyngeal, esophageal, pancreatic, urothelial, skin, and hematological cancers (7). Functionally, B7-H1 overexpression correlates with worse prognosis and resistance to anti-cancer therapies (8–11). Mechanistically, B7-H1 binds to its receptor, programmed death 1 (PD-1, also known as CD279) on T cells, inducing T-cell apoptosis and thus protecting tumor cells from immune attack (12). In return, B7-H1 can also induce an anti-apoptotic signal in the tumor cells in response to a signal from PD-1, and can promote resistance against T-cell-mediated killing (13).

The significance of B7-H1/PD-1 signaling in maintaining an immunosuppressive tumor microenvironment makes this protein a promising target for anti-cancer therapies. In this study, we examined the expression of B7-H1 in two different lung carcinoma cells, assessed the significance of tumor-associated B7-H1 in T-cell proliferation, and evaluated the impact of B7-H1 blocking antibody on tumor growth in a xenograft tumor model.

## Material and Methods

### Cell lines and experimental animals

All protocols using human or animal samples were approved by the Ethics Committee of Soochow University (Suzhou, China; Approval No. 2013-072).

Human adenocarcinoma cells A549 and the mouse Lewis lung carcinoma (LLC) cells were purchased from the Cell Bank of Chinese Academy of Sciences (Shanghai, China). The A549 cells were cultured in Dulbecco's Modified Eagle's Medium (Invitrogen, USA) supplemented with 12.5% fetal bovine serum (FBS; Invitrogen). The LLC cells were cultured in RPMI1640 medium (Invitrogen) supplemented with 12.5% FBS. All cells were incubated in a sterile incubator at 37°C with 5% CO_2_.

Wild-type C57Bl/6 mice (8 weeks old) were purchased from Shanghai Laboratory Animal Center (China). All mice were housed in a specific pathogen-free facility at room temperature of 22±1°C on a 12-h light/dark cycle with access to food and water *ad libitum*.

### Flow cytometry

Flow cytometric analysis was performed using standard protocols on either *in vitro* cultured cells or cells isolated from mouse tissues (see below). For *in vitro* cultured cells, the cells were detached using 0.25% EDTA (Invitrogen; for *in vitro* cultured cells) and washed twice with phosphate-buffered saline (PBS). To prepare single-cell suspensions from mouse tumors, we removed the xenograft tumor tissues from the mice, cut it into small pieces with sterile scissors, and digested the tissue pieces with dissociation solution [RPMI medium supplemented with 5% FBS, collagenase type I (200 U/mL), and DNase I (100 μg/mL)] for 30 min at 37°C, with repeated pipetting and vortexing every 10 min during incubation. Following incubation, the cell suspension was passed through a 70-µm cell strainer and washed twice with PBS. For preparation of a single-cell suspension from mouse spleen, the spleen was dissected, pressed into single cells under the pressure of the plunger of a 3-mL syringe through a 70-µm cell strainer, and washed twice with PBS. The cells isolated from either tumor tissues or spleen were then treated with red blood cell lysis buffer (15.5 mM NH_4_Cl, 10 mM KHCO_3_, 10 µM EDTA) and washed twice with PBS. The cells were then incubated with the proper fluorophore-conjugated antibodies at 4°C in dark for 30 min, washed three times with PBS, and examined on a flow cytometer (Cytomics FC 500, Beckman Coulter, USA), with a total of 50,000 events collected for each sample. The following antibodies were purchased from Biolegend (USA) and used in flow cytometry analyses: phycoerythrin (PE)-conjugated anti-human and mouse B7-H1, PE-Cy5-conjugated anti-CD3, and PE-Cy7-conjugated anti-CD45. Flow cytometry analysis was performed using FlowJo software (FlowJo, USA).

### *In vitro* T-cell proliferation assay

Whole blood was collected from healthy individuals at the Suzhou Blood Center (Suzhou, China) and subjected to density gradient separation on Ficoll-Paque Plus (GE Healthcare, USA). After centrifugation, the peripheral blood mononuclear cell (PBMC) layer was collected, seeded onto a tissue culture plate, and incubated at 37°C in a 5%-CO_2_ incubator. After 2-h incubation, cells in suspension were collected following gentle pipetting the medium, and these were predominantly T cells. The isolated T cells were labeled with carboxyfluorescein succinimidyl ester (CFSE; Biolegend) as previously described (14). Meanwhile, A549 cells were treated with cisplatin (25 mg/mL; Biolegend) for 3 h. The CFSE-labeled T cells were then seeded into 96-well plates (2×10^5^ cells/well) that had been pre-coated overnight with anti-CD3 (5 µg/mL, Biolegend) and anti-CD28 (2.5 µg/mL, Biolegend) at 4°C. The cisplatin-treated A549 cells with or without B7-H1 blocking antibody (50 µg/mL, Biolegend) were then added to CFSE-labeled T cells at a T:A549 ratio of 1:2, 1:4, or 1:8. Each condition was tested in triplicate. After 72 h, all cells were collected and T-cell proliferation was examined by flow cytometry.

### *In vivo* xenograft model

The experimental mice were divided into three groups (n=5/group), i.e., negative control (NC), LLC-injected (LLC), and LLC+anti-B7-H1 (anti-B7-H1) groups. For mice in the LLC and anti-B7-H1 groups, the xenograft tumor model was established by subcutaneously injecting LLC cells (2×10^6^/mouse) into the inguinal region on day 1. The mice in NC group received an equal-volume PBS injection. Starting from day 5, mice in the anti-B7-H1 group received intravenous injection of anti-B7-H1 antibody (Biolegend; 50 µg/mouse) every 5 days until day 30, whereas mice in the NC or LLC group received vehicle (PBS) injection following the same schedule. Tumor growth was monitored every 5 days with the tumor area calculated as V=1/2×a×b^2^, where ‘a' is the length and ‘b' is the width of the tumor.

### Statistical analysis

All *in vitro* experiments were repeated independently at least three times. Statistical analysis was performed using GraphPad Prism5 software (GraphPad Software, USA). All data are reported as means±SD and compared using analysis of variance (ANOVA). A P value of <0.05 was considered to be statistically significant.

## Results

### B7-H1 was expressed in A549 lung cancer and LLC cells

To assess the involvement of tumor-associated B7-H1 in lung cancer development, we first measured its expression level in two distinct lung carcinoma cell lines: human A549 cells and mouse LLC cells. By flow cytom-etry, we found that B7-H1 was abundantly expressed in both cell lines, with positivity among A549 cells (55.9±2.4%) and positivity among LLC cells (68.0±1.3%). The gating for B7-H1+ cells is shown in Supplementary Figure S1.

### A549-associated B7-H1 essentially inhibited T-cell proliferation *in vitro*

**Figure 1.**
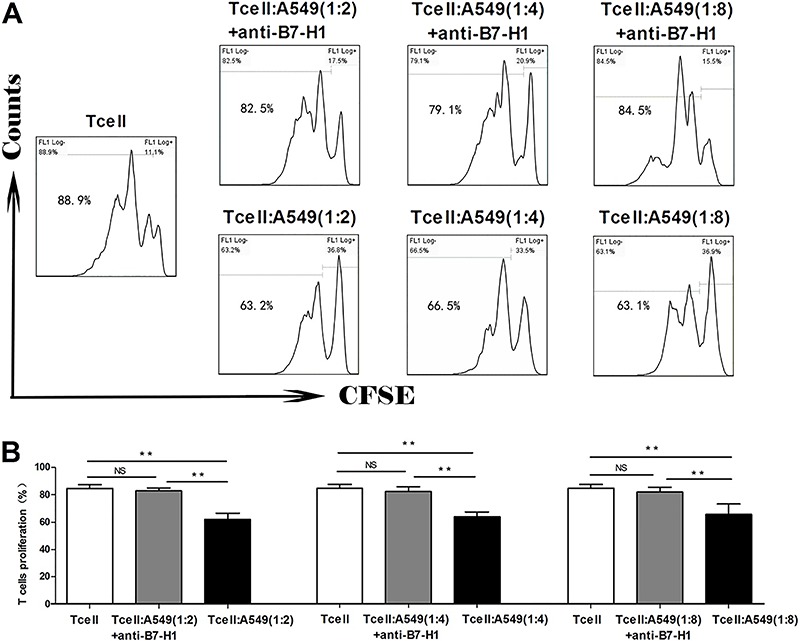
*A*, Representative flow cytometry graphs for each experimental group, with the percentage of proliferating T cells (cells left to the first peak of carboxyfluorescein succinimidyl ester (CFSE)+ cells) labeled in the graph. *B*, Quantitative analysis of proliferating T cells among different groups. Tumor-associated B7-H1 is essential for regulating T-cell proliferation. T cells isolated from human peripheral blood mononuclear cells were labeled with CFSE, stimulated with anti-CD3 and anti-CD28, and co-cultured with T cells alone or with A549 cells at the T:A549 ratios of 1:2, 1:4 or 1:8, in the absence (+A549) or presence of anti-B7-H1 (+anti-B7-H1). T-cell proliferation was examined by flow cytometry after 72 h. NS: non-significant. **P<0.01 (ANOVA).

To examine the biological significance of tumor-associated B7-H1 in cancer immunity, we co-cultured CFSE-labeled T cells isolated from human PBMCs alone (T cells only) or with different numbers of A549 cells in the absence (1:2 +A549, 1:4 + A549, or 1:8 + A549) or presence of anti-B7-H1 antibody (1:2 +anti-B7-H1, 1:4 + anti-B7-H1, or 1:8 + anti-B7-H1). To stimulate the basal-level T-cell proliferation, we added anti-CD3 and anti-CD28 into all experimental groups, and T cells treated with only anti-CD3 and anti-CD28 (T cells) were used as controls (15). By gating for T cells (Supplementary Figure S2), we found that *in vitro* T-cell proliferation was significantly inhibited with the addition of A549 cells into to the co-culture system (P<0.01 for 1:2 + A549, 1:4 + A549, or 1:8 + A549 groups compared with the control T cell group), although no dramatic differences were observed with different number of A549 cells added (P*>*0.05 compared with 1:2 + A549, 1:4 + A549 or 1:8 + A549 groups). The addition of anti-B7-H1, however, markedly reversed the inhibition of T-cell proliferation exerted by A549 cells, i.e., no significant difference in T-cell proliferation was detected between the control T cell group and the anti-B7-H1 (1:2, 1:4 or 1:8) groups (P>0.05; Figure 1).

### *In vivo* administration of anti-B7-H1 antibody was associated with markedly reduced tumor growth

**Figure 2.**
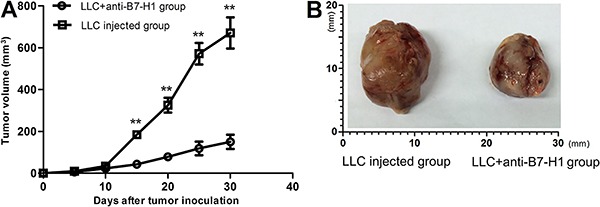
Anti-B7-H1 antibody inhibition of tumor growth *in vivo.* Lewis lung carcinoma (LLC) cells were subcutaneously injected into C57BL/6 mice to form xenograft tumors. Anti-B7-H1 or vehicle control (LLC injected group) was administered intravenously every 5 days thereafter. *A*, Tumor growth was monitored every 5 days by calculating tumor area, which was compared between the anti-B7-H1 and control groups. **P<0.01, compared with the control group (ANOVA). *B*, Representative images of xenograft tumors on day 30 after LLC injection in the control and anti-B7-H1 groups.

For *in vivo* analysis, we established a xenograft model using LLC cells and assessed the impact of B7-H1 on tumor growth by intravenous administration of anti-B7-H1 antibody. As shown in Figure 2, anti-B7-H1 administration significantly inhibited the tumor growth starting from day 15 after injection (Figure 2A) and led to much smaller tumors by day 30 (Figure 2B).

### *In vivo* administration of anti-B7-H1 antibody significantly increased T-cell numbers in the spleen

**Figure 3.**
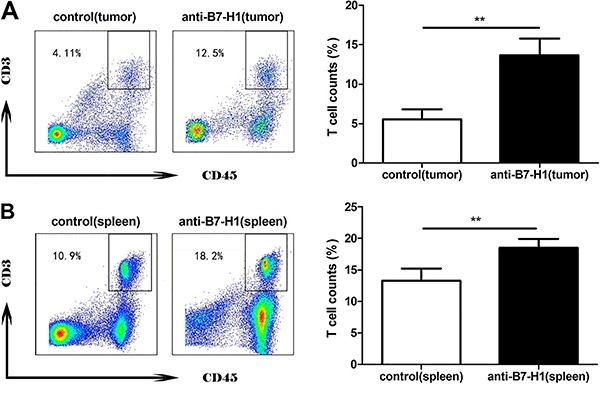
Representative flow graphs (*left*) and results of quantitative analysis (*right*). B7-H1-induced anti-tumorigenesis is associated with a significantly higher number of CD3+CD45+ T cells in both xenograft tumors and spleens. Xenograft tumors (*A*) and mouse spleens (*B*) were isolated from either control or anti-B7-H1 mice, and digested into single-cell suspensions. The CD3+CD45+ T-cell population was examined by flow cytometry. **P<0.01, compared with the control group (*t*-test).

To explore the potential involvement of T-cell proliferation in the anti-tumorigenic effect as observed in Figure 3, we quantified the CD3^+^CD45^+^ T-cell population within both tumors and spleens by flow cytometry. There were significantly higher percentages of CD3^+^CD45^+^ T cells in both the tumors and spleens of mice that received anti-B7-H1 treatment (P<0.01 compared with mice bearing xenograft tumors but not receiving anti-B7-H1 treatment; Figure 3).

## Discussion

In this study, we showed that B7-H1 was highly expressed in two lung carcinoma cell lines: the human A549 cell line and mouse LLC cells. *In vitro,* the tumor-associated B7-H1 was essential for suppressing T-cell proliferation. *In vivo,* targeting by B7-H1 led to a significant reduction in tumor growth, which was associated with a higher number of total T cells in both the tumor tissue and spleen.

B7-H1 was first identified "by searching for molecules that share homology with the immunoglobulin V and C domains of B7-1 and B7-2 among the human cDNA expressed sequence tags in the National Center for Biotechnology Information database" (16). B7-H1 mRNA is abundantly detected in multiple tissues including heart, skeletal muscle, placenta, and lung tissues; weakly expressed in thymus, spleen, kidney, and liver tissues; and not detected in brain, colon, small intestine, or PBMCs (16). However, B7-H1 protein is only marginally expressed in most normal tissues and significantly up-regulated in various tissues in response to inflammatory cytokines (17–21). In most cancer cells, B7-H1 protein expression is highly up-regulated (7). In this study, we detected B7-H1 expression in more than 55% of A549 cells and almost 70% of LLC cells, consistent with its abundant expression in human lung cancer reported in previous studies (22–24).

The abundant expression of B7-H1 in human lung cancers suggests that B7-H1 is functionally important for the development of lung cancer, and thus, targeting this molecule may provide therapeutic benefits for cancer treatment. Consistently, antibodies blocking/targeting B7-H1 have demonstrated promising efficacy in clinical trials (25). However, the biological activities of B7-H1 in lung cancer are not completely understood, and most have been deduced from the findings in other human cancers. To address this issue, we explored the biological activities of B7-H1 in both human lung carcinoma A549 and mouse Lewis lung carcinoma LLC cells.

Accumulative studies show that B7-H1 presents both co-stimulatory and co-inhibitory activities (26–28). The co-inhibitory activity is mainly mediated through the receptor PD-1 expressed on activated CD4^+^ and CD8^+^ T cells, B cells, Tregs, natural killer cells, and other tumor-infiltrating lymphocytes (29). Through interaction with PD-1, B7-H1 promotes T-cell apoptosis (30,31), impairing inflammatory cytokine production and inhibiting T-cell proliferation (32 [Bibr B33] 34). In addition, B7-H1/PD-1 interaction can also increase the Foxp3^+^ immunosuppressive Tregs within the tumor microenvironment (35,36). Although the molecular mechanisms downstream of PD-1 are not fully understood, B7-H1/PD-1 interaction recruits the Src homology region 2 domain containing phosphatases 1 and 2 (SHP-1, SHP-2) and inhibits TCR signaling (37). In addition to PD-1, B7-H1 may also signal through CD80 to inhibit T-cell responses (38). The co-stimulatory action of B7-H1 is independent of PD-1 and mediated through an unidentified receptor (12,16,39). Furthermore, B7-H1 may function as a receptor to transmit anti-apoptotic signals into cancer cells (13). In this study, we showed that co-culturing of the B7-H1-expressing A549 cells with T cells *in vitro* resulted in inhibition of T-cell proliferation, and the addition of B7-H1 blocking antibody markedly relieved the inhibition of T-cell proliferation. These data support the co-inhibitory activity of B7-H1 in lung cancer. In the *in vivo* xenograft tumor model, although we did not measure T-cell proliferation directly, we observed a dramatic increase in total T cells in both tumor tissues and spleen following anti-B7-H1 treatment, suggesting that B7-H1 controls total T-cell population locally (within the tumor microenvironment) and distantly (in the spleen).

The co-inhibitory activities of B7-H1/PD-1 signaling represents an ideal target for cancer immunotherapy. Multiple strategies targeting B7-H1 and/or PD-1 have been developed and tested in animal models or clinical trials (7,40). Consistently, we also examined the effects of B7-H1 blocking antibody on tumor growth in the xenograft mouse model. We found that B7-H1-expressing LLC cells led to aggressive tumor growth *in vivo,* which was significantly inhibited by anti-B7-H1 treatment, supporting the anti-cancer efficacy of targeting B7-H1.

In summary, this study provides novel evidence for the co-inhibitory activity of B7-H1 in lung cancer. This action is at least mediated through the regulation of T-cell proliferation. Blocking B7-H1 offers a promising strategy for anticancer therapy. Although we showed the anti-tumor activity of B7-H1 blocking antibody, we noticed that the tumor growth was not completely stopped, but only modestly inhibited by targeting B7-H1, suggesting that B7-H1 is not the only molecule that mediates immune inhibition. Therefore, it should be combined with other anticancer approaches to achieve a more robust effect.

## Supplementary material

Click here to view [http://bjournal.com.br/supplementary_material/5263.pdf].
